# Academic Mentoring for Psychiatric Trainees During the Pandemic

**DOI:** 10.1192/j.eurpsy.2022.194

**Published:** 2022-09-01

**Authors:** M. Rojnic Kuzman

**Affiliations:** University Hospital Center Zagreb, University of Zagreb, Department Of Psychiatry And Psychological Medicine, Zagreb School Of Medicine, Zagreb, Croatia

## Abstract

In most academic settings, mentoring students is a part of the activities of the academic staff. Usually, associate professors, assistant professors and professors are required to mentor graduate and postgraduate students, and in some academic settings postgraduate students are required to mentor junior students. In psychiatric clinical settings, mentoring also extends to the supervision and evaluation of clinical work of postgraduate psychiatric trainees, usually for different parts of the psychiatric training. Depending on the local organisation of academic and clinical work, numbers of mentees per one mentor as well as description of activities may vary greatly across regions. Nevertheless, “mentoring” is not systematically taught and evaluated in the majority of systems but is infrequently self-taught. The situation of COVID-19 pandemic, especially in the first wave, has created a new situation which needed quick adaptations in many of the fields of the academic work, mentoring included. In this presentation, these new situations and lessons learnt will be presented and discussed from the point of the academic centre in Croatia.

**Disclosure:**

No significant relationships.

